# Density functional theory analysis of the structural, electronic, elastic, phonon dispersion and AIMD properties of KMgX_3_ (X = O, S, Se)

**DOI:** 10.1039/d5ra04332h

**Published:** 2025-08-18

**Authors:** Mazhar Haleem Awan, Sehrish Munsif, Huma Habib

**Affiliations:** a Department of Physics, Abbottabad University of Science and Technology Abbottabad KPK Pakistan mazharhaleem7@gmail.com +923149888807; b CAS Key Laboratory of Science and Technology on Applied Catalysis, Dalian Institute of Chemical Physics, Chinese Academy of Science Dalian 116023 China sehrishmunsifdicp@gmail.com; c Center for Micro and Nano devices (CMND), Comsats University Islamabad Pakistan huma.habib@comsats.edu.pk

## Abstract

Perovskite chalcogenides have attracted significant interest due to their potential applications in optoelectronics, catalysis, and renewable energy systems. This paper examines the structural, electronic, elastic, and phononic properties of KMgX_3_ (X = O, S, Se) using density functional theory (DFT) in the context of the full-potential linearized augmented plane wave plus local orbital (FP-LAPW + lo) approach. Their stability in the cubic phase (*Pm*3̄*m* symmetry) is confirmed by the computed lattice parameters for KMgO_3_ (4.1325 Å), KMgS_3_ (5.0008 Å), and KMgSe_3_ (5.2070 Å). KMgO_3_ exhibits semiconducting behavior with a direct bandgap of 7.323 eV in the spin-up state, according to electronic band structure studies, whereas KMgS_3_ and KMgSe_3_ show metallic properties. Elastic constants (C_11_, C_12_, and C_44_) meet the requirements for mechanical stability, which is evaluated using the Born criterion. Upon further examination of mechanical characteristics such as Bulk modulus, Shear modulus, Young's modulus, and Poisson's ratio. Materials such as KMgO_3_ and KMgS_3_ exhibit ductile behavior, whereas KMgSe_3_ exhibits brittleness. Phonon dispersion curves and *ab initio* molecular dynamics simulations confirm the dynamical and thermal stability of these compounds. The results show that KMgX_3_ perovskites have potential uses in optoelectronic devices and spintronics.

## Abstract 2

Perovskite chalcogenides have attracted significant interest due to their potential applications in optoelectronics, catalysis, and renewable energy systems.

## Graphical abstract

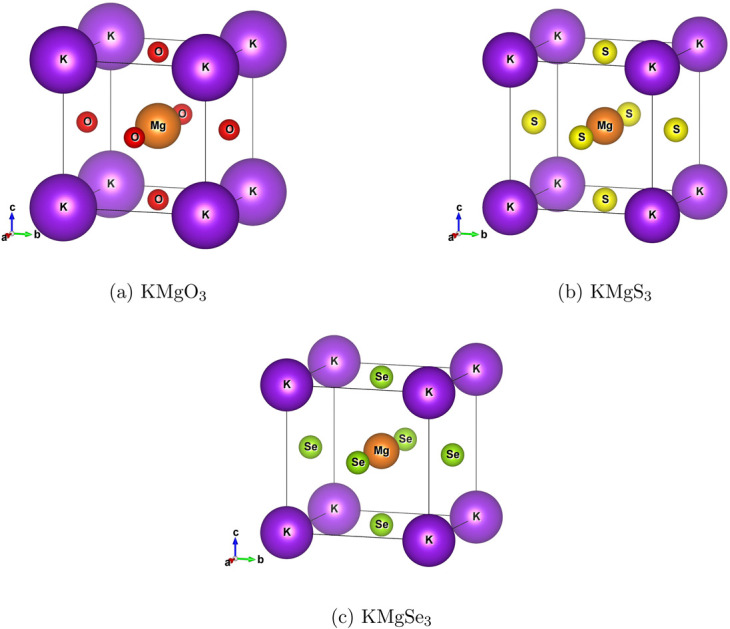

## 1 Introduction

Ternary alkaline-earth chalcogenides have recently attracted considerable attention due to their promising physical properties and potential applications in optoelectronic and spintronic devices. Among these, compounds of the general formula ABX_3_, where A and B are alkaline or alkaline-earth metals and X is a chalcogen (O, S, Se), have demonstrated tunable band gaps, stable crystal structures, and interesting elastic and vibrational behaviors depending on the elemental composition.^1–3^ Moreover, studies have explored a variety of perovskite-like and non-perovskite ABX_3_ materials; for instance, the structural and electronic characteristics of alkali-metal chalcogenides such as NaMgX_3_ and LiMgX_3_ have been reported using density functional theory (DFT) approaches.^4,5^ These studies emphasize their potential for ultraviolet (UV) optoelectronic applications, attributed to their wide band gaps and chemical stability. Ternary metal chalcogenides, particularly those of the form AMgX_3_ (A = alkali/alkaline earth metals; X = O, S, Se), have emerged as promising candidates for next-generation optoelectronic and spintronic devices due to their tunable band gaps, structural versatility, favourable mechanical and vibrational properties. Among them, materials with perovskite-like or distorted perovskite structures have gained significant interest, especially in the context of wide-bandgap semiconductors and non-toxic, earth-abundant alternatives to conventional materials.^6^ Theoretical study revealed that the compound such as CaMgX_3_, have good mechanical stability, thermal behavior, and electronic structure.^7^

Specifically, perovskite oxides with the general formula ABO_3_ have garnered significant attention due to their diverse range of physical properties, which are highly sensitive to the choice of A and B-site cations. Depending on the elemental composition, these materials can exhibit phenomena such as antiferromagnetism (*e.g.*, LaTiO_3_),^8^ ferroelectricity (*e.g.*, BaTiO_3_),^9^ ferroelasticity (*e.g.*, SrTiO_3_),^10^ antiferroelectricity (*e.g.*, PbZrO_3_),^11^ and ferromagnetism (*e.g.*, YTiO_3_).^12^ Recent research has increasingly focused on exploring the magnetic and electronic properties of these materials through both experimental and theoretical approaches. It was reported that PrMnO_3_, in its cubic perovskite phase, exhibits half-metallic behaviour. Similarly, it was predicted that BaFeO_3_ (BFO) can become a half-metallic ferromagnet under the influence of strain and electronic correlation, using density functional theory (DFT). Moreover, it was demonstrated that CaFeO_3_ exhibits half-metallicity at equilibrium, but upon applying compressive strain beyond a critical lattice constant, the material undergoes a sudden transition to a metallic state, accompanied by a loss of integer magnetic moment. In a more recent study BaNpO_3_ was studied and found to be a half-metallic ferromagnet using the modified Becke–Johnson (mBJ-GGA) potential and the Hubbard U correction (GGA + *U*). Moreover, some perovskite oxides, such as LiBeO_3_ (ref. 13) and KMgO_3_,^14^ have been identified as half-metallic ferromagnets, making them promising candidates for spintronic applications. In a comprehensive density functional theory (DFT) study it was demonstrated that KMgO_3_ is thermodynamically stable in the ferromagnetic (FM) configuration, with a calculated formation energy of −0.075 eV.^15^ Importantly, they reported a high Curie temperature *T*_c_ of 560.4 K, a feature attributed to the polarization of O-2p orbitals which contributed to a robust magnetic moment of 3 μB, retained under pressures up to 116.2 GPa. These results suggest that KMgO_3_ possesses both mechanical and magnetic robustness, making it an excellent candidate for spintronic applications. In a separate study, it was investigated that hydrogen-substituted KMgO_3−*x*_H_*x*_, demonstrating its potential as a viable hydrogen storage material, further broadening the application range of this compound.^16^

Traditionally, half-metallic ferromagnets (HMFs), which exhibit metallic behavior for one spin channel and semiconducting behavior for the other, have been based on transition metals, owing to their partially filled d or f orbitals, which are essential for magnetic behavior. However, recent experimental and theoretical advances have shown that transition-metal-free compounds composed of light elements (*e.g.*, alkali and alkaline-earth metals) can also exhibit half-metallic ferromagnetism. Notably, compounds such as LiBeO_3_ and KBeO_3_, especially when combined with light anions like carbon, nitrogen, or oxygen, have demonstrated spin polarization arising from s and p orbital interactions, rather than from conventional d and f orbitals. These discoveries open new avenues for spintronic device development that avoids reliance on heavy or rare elements. Furthermore, prior work has seldom addressed the combined analysis of structural, electronic, elastic, phonon dispersion, and thermal stability through *ab initio* molecular dynamics (AIMD), which is crucial for understanding real-world device performance. Additionally, previous works often overlook the implications of these materials for spintronic applications, despite their non-magnetic semiconducting nature and potential for spin–orbit coupling enhancement when heavier chalcogens are introduced.^17–19^ Therefore, a comprehensive DFT-based analysis that encompasses structural, electronic, vibrational, and thermodynamic properties of KMgX_3_ compounds is necessary to bridge this knowledge gap.

This study addresses that need by presenting a systematic and comparative first-principles investigation of KMgX_3_ (X = O, S, Se), analysing their mechanical stability, band structures, phonon behavior, and thermal resilience. The results provide new insights into how the choice of chalcogen affects the properties of these materials and their suitability for optoelectronic and spintronic applications.

## 2 Computational methods

Physical characteristics of KMgX_3_(X = O, S and Se) compounds were investigated using the WIEN2k technique under the framework of density functional theory (DFT). Whole calculations were performed using the full-potential linearized augmented plane wave plus local orbitals (FP-LAPW + lo) approach.^20,21^ The WC-GGA method is used along with TB-mBJ hybrid approximation calculations.^22^ The necessary initial parameters, such as cutoff energy, lattice parameters, and Brillouin zone (BZ) grid, were present in the input data. The smaller radius of muffin-tin (MT) sphere and the larger the value of reciprocal grating vector (RMT × KMAX) are multiplied because of, to determine the cutoff energy in the cutoff plane wave, which is taken as −6.0 Ry (Rydberg). In addition, the partial waves employed inside the sphere have a maximum value of *l*_max_ = 10 and *G*_max_ = 12 au^−1^ was selected as the maximum vector magnitude of the Fourier series charge density expansion. To find better results, we used a finer kmesh grid of 10 × 10 × 10 and a denser 12 × 12 × 12 over the Brillouin zone, which was used for the computations to calculate the structural and other properties. Energy and charge convergence criteria are typically defined as 10^−4^ Ry, and TETRA smearing is used. To find out the elastic constant, an energy strain approach is included in the WIEN2k code and by subjecting the cubic lattice to deformation, only three independent elastic constants *C*_11_, *C*_12_, and *C*_44_ can be calculated.^23,24^ The phonon dispersion properties of these compounds are computed using the Parlinski–Li–Kawazoe method, called PHONOPY. Lattice dynamics simulations utilize the PHONOPY code in conjuction with the WIEN2k open-source program, which was used to calculate the forces. Using the 2 × 1 × 1 supercell and the finite displacement method, a smaller *k*-point mesh (1 × 3 × 3) was made to determine the phonon dispersion.^25^

## 3 Results and discussions

### 3.1 Structural properties

**Fig. 1:**
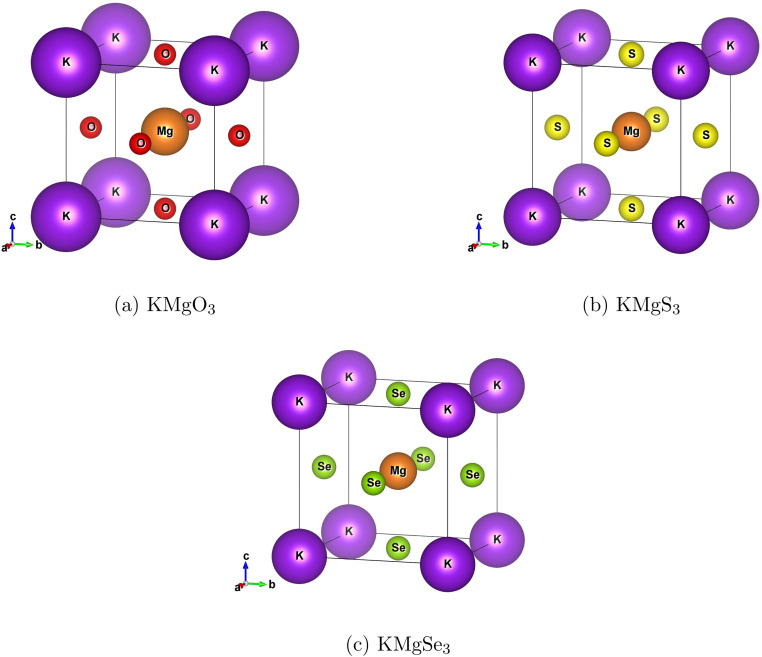
Structural views of KMgX_3_(X = O, S and Se) chalcogenides perovskite: (a) KMgO_3_, (b) KMgS_3_, and (c) KMgSe_3_.

KMgX_3_ (X = O, S and Se) are tenary alkali-earth metal chalcogenides perovskite compounds having the space group of *Pm*3̄*m* with the international number of 221, and their fractional coordinates revealed the unit cells. The structural equation of ABX_3_ is the compound where ‘A = K’ atom is located at the cube corner of 1-a Wyckoff site coordinates (0, 0, 0), the 1-b Wyckoff site is occupied at body position (0.5, 0.5, 0.5) which is designates as ‘B = Mg’ atom. Lastly, the 3-c Wyckoff site (0, 0.5, 0.5) is chalcogenides ‘X’ anion (O, S and Se) occupied at face-centered locations in cubic crystal structure. Fig. 1(a, b and c) shows that five atoms are placed in different arrangements of the elements, which are visualized using the VESTA.^26^ To optimize lattice constants were used to examine the structural properties of KMgX_3_ and the *E*–*V* curves for NM (non-magnetic), FM (ferromagnetic), AFM (antiferromagnetic) and FSM (Fixed Spin moment) are obtained by using the Birch–Murnaghan equation of state (EOS),^27^ which represents the structure stability.

**Fig. 2:**
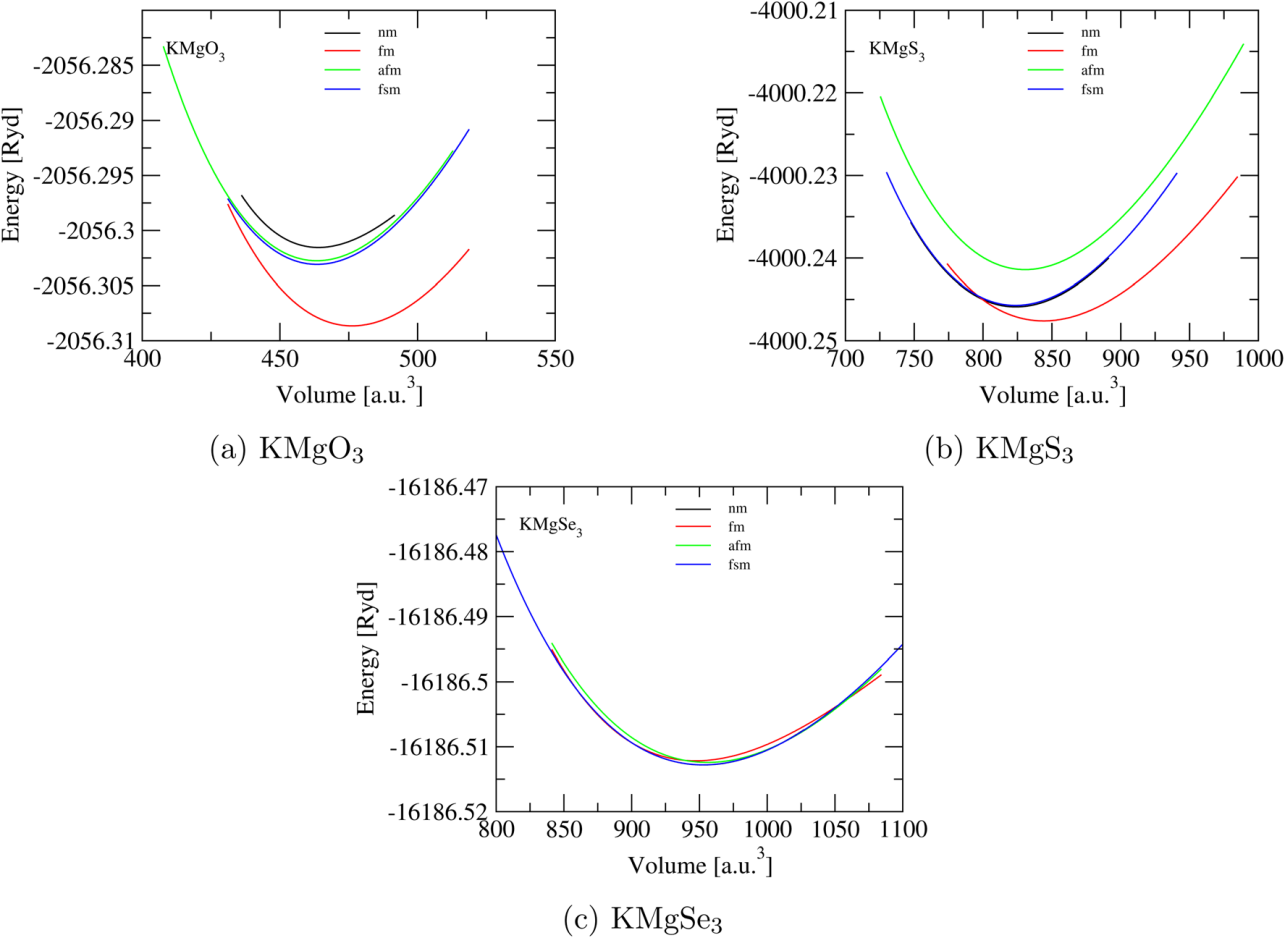
*E*–*V* curves of KMgX_3_ (X = O, S and Se) compounds which correspond to minimal energy point: (a) KMgO_3_, (b) KMgS_3_, and (c) KMgSe_3_.

In Fig. 2(a) FM (red curve) is stable at minimal energy −2056.31 (Ryd), Fig. 2(b) FM (red curve) is stable at minimal energy −4000.25 (Ryd) and finally, Fig. 2(c) FSM (blue curve) is stable at minimal energy near to −16186.52 (Ryd). The WC-GGA approximation calculates minimized forces, and the system was unfastened until the forces acting on each atoms of the unit cell were negligible. Equation of Birch–Murnaghan (EOS)^27^ reveals that all calculated values of lattice constants listed in Table 1 are in good agreement with other available data.^28^eqn (1) includes *B*_0_, *B*′, *V*_0_, and *E*(*V*), which are the bulk modulus, the first derivative of unit cell volume, and energy at equilibrium ground states, respectively.1
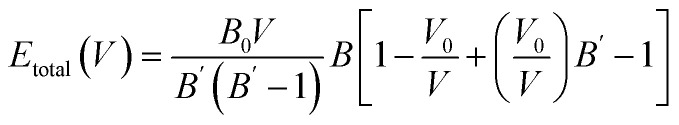


**Table 1:** Computational calculated values for KMgX_3_ (X = O, S and Se) compounds

Compound	*a* (Å), others	*V*_o_ (A^3^)	*B*(GPa)	*B*′	*E*_o_
KMgO_3_	4.1325, 4.1344 (ref. 28)	476.24	63.095	4.55	−2056.399
KMgS_3_	5.0008, —	843.97	29.454	4.84	−4000.247
KMgSe_3_	5.2070, —	952.72	31.013	4.41	−16186.513

### 3.2 Band structure

**Fig. 3:**
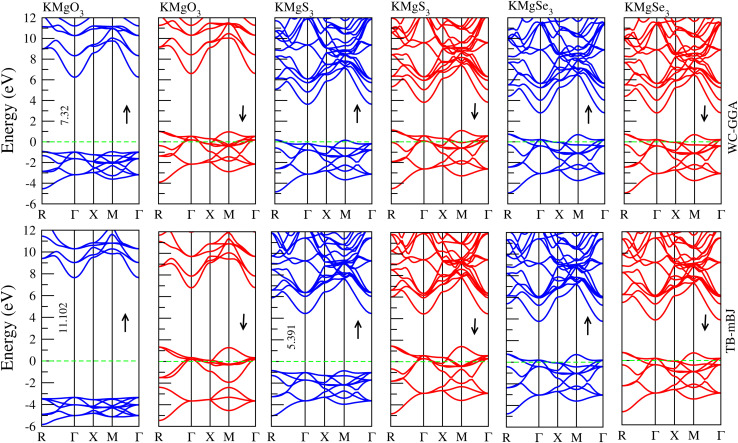
Present the band structure of KMgX_3_(X = O, S and Se) with WC-GGA.

Depending on their distinguishing characteristics, materials can be categorized as metallic or semiconducting using electronic band structures, which offer important insights into the physical behavior of solids. Band structure is determined by the WC-GGA exchange correlation potential method along with TB-mBJ potential. The high symmetry orientations, *i.e.* R, Γ, X, M,Γ points in the first Brillioun zone for KMgX_3_(X = O, S and Se) cubic phase perovskites. Fig. 3 displays the band structure of KMgX_3_(X = O, S and Se) compounds, where the blue color shows spin-up and the red shows spin-down states. According to Fig. 3, the bandgap is noted between the minima and maxima of the conduction and valence bands at the Γ point along the symmetry axis. Therefore, these materials have a direct bandgap transition along the Γ–Γ direction, and this confirms that the semiconductor behavior of only one compound is KMgO_3_ for spin-ups having a gap of 7.323 eV closer to the Fermi level (literature value = 7.15 eV^28^), and empty states (holes) are created just above the valence band. It can be concluded from the calculated values that KMgO_3_ is a half-metal with p-type character in the semiconducting spin channel, means that the material behaves like a metal for electrons of one spin direction and like a semiconductor or insulator for electrons of the opposite spin direction. This property makes them 100% spin-polarized at the Fermi level, which is very useful in spintronics, where the spin of electrons is used for information processing instead of charge. KMgO_3_ is an unusual example of a half-metallic material because it does not contain transition metals, which are typically responsible for magnetism in conventional half-metallic ferromagnets due to their partially filled d and f orbitals. In KMgO_3_, one spin channel exhibits metallic behavior, allowing electrons to conduct freely, while the opposite spin channel displays semiconducting behavior. Notably, this semiconducting spin channel has p-type character, meaning that the conduction is dominated by holes rather than electrons. This implies that the Fermi level lies closer to the valence band, and electrical conduction in this spin channel would occur through the movement of these positively charged holes. The magnetic and half-metallic properties in KMgO_3_ are believed to originate from p-orbital electrons, particularly those of oxygen, rather than the d-orbitals seen in traditional systems. This makes KMgO_3_ part of a growing class of “d^°^” or p-orbital magnetic materials, which are significant because they challenge the conventional understanding of magnetism and open new possibilities for spintronic materials based on lighter, more abundant elements. All the others are metals, which are listed in Table 2. The transition from semiconducting to metallic behavior in KMgX_3_(X = O, S, Se) is primarily driven by increased orbital overlap and band structure modifications as the chalcogen atomic size increases. This leads to narrower or closed band gaps in KMgS_3_ and KMgSe_3_, in contrast to the wider band gap of KMgO_3_, thereby explaining the observed shift in electronic behavior.

**Table 2:** Provides the bandgap with WC-GGA potential of KMgX_3_(X = O, S, Se)

Compound	WC-GGA(↑)	TB-mBJ(↑)	Others	WC-GGA(↓)	TB-mBJ(↓)
KMgO_3_	7.323	11.102	7.15 (ref. 25)	0.0	0.0
KMgS_3_	0.0	5.391	—	0.0	0.0
KMgSe_3_	0.0	0.0	—	0.0	0.0

The ionic radius increases significantly from O^2−^ to Se^2−^. This leads to larger and more diffuse p orbitals in S and Se compared to O. The overlap between the Mg 3s/3p orbitals and the chalcogen p orbitals becomes more pronounced with the larger anions (S and Se), which promotes wider energy bands. KMgO_3_, the energy difference between the valence band (dominated by O-2p states) and the conduction band (mainly Mg states) is significant enough to produce a semiconducting gap. However, in KMgS_3_ and KMgSe_3_, the increased orbital overlap leads to band broadening, which can cause the valence and conduction bands to overlap or touch, effectively closing the band gap and resulting in metallic conductivity. The electronegativity of the chalcogen decreases from O to Se. Lower electronegativity in Se compared to O results in less localization of the valence electrons, which also contributes to band broadening and the delocalization of charge carriers, favoring metallicity. Moreover, larger anions distort the local bonding environment differently, which can impact crystal field splitting and modify the band edges. These structural changes may further promote metallic behavior in the heavier analogs.

There is a significant difference in band gap values between the WC-GGA and TB-mBJ methods particularly in the case of KMgS_3_. This is due to the intrinsic limitations of the WC-GGA functional in accurately describing the electronic structure of semiconductors and insulators. WC-GGA tends to underestimate band gaps, which is a well-known issue with standard GGA-based functionals. In contrast, the TB-mBJ method includes a potential that better mimics the behavior of the exact exchange potential, especially near the band edges, leading to more accurate predictions of electronic band gaps. For KMgS_3_, this results in a noticeably larger band gap for spin up calculations when calculated with TB-mBJ compared to WC-GGA. The author uses this difference to highlight the importance of choosing appropriate functionals when studying electronic properties and emphasizes that TB-mBJ provides a more reliable description of the material's semiconducting behavior.

### 3.3 Density of states and partial density of states (DOS & PDOS)

**Fig. 4:**
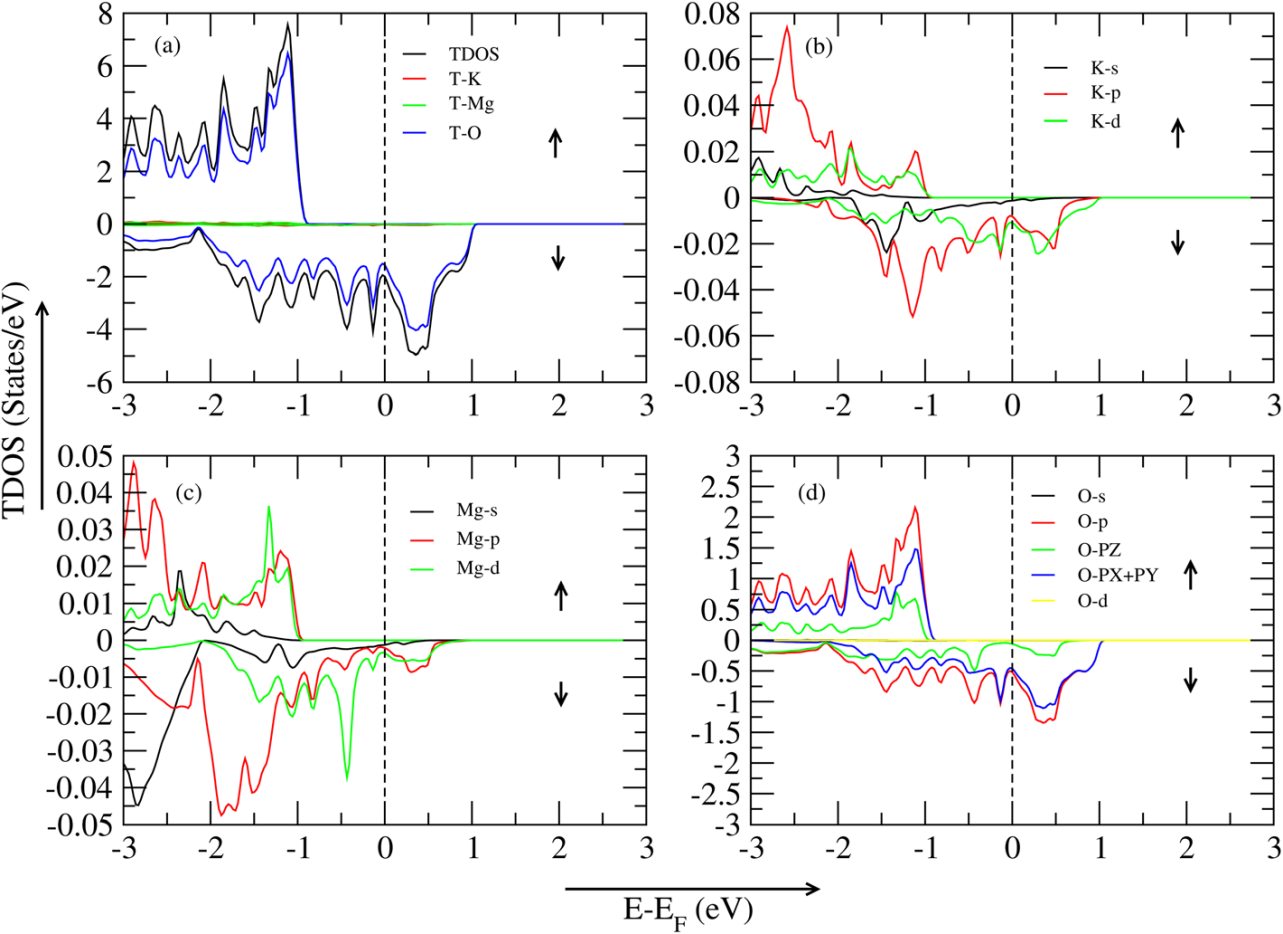
TDOS and PDOS of the KMgO_3_ compound with WC–GGA: (a) TDOS, (b) PDOS for K atoms, (c) PDOS for Mg atoms, and (d) PDOS for O atoms.

In this section, all graphs are taken by the WC-GGA approximation method. Fig. 4(a) represents the TDOS (total density of states) of the KMgO_3_ compound. The graph is separated into two parts: spin-up and spin-down states. In the spin-up region, the significant contribution of TDOS (KMgO_3_) to 7.32 eV, and secondly, the oxygen atom shows an active role in this region at around 6.24 eV of VBM (Valence Band Maxima). The other part is the CBM (Conduction Band Minima), a huge bandgap of 7.323 eV show a semiconductor nature. In the spin-down region, there is no bandgap difference between them, so the material shows a metallic nature. In Fig. 4 (b), spin-up active role play is the K-p hybrid ortibal near the Fermi level of 0.019 eV in VBM, and in the other part, CBM, a huge bandgap difference shows them, and spin-down is the metallic state. Fig. 4(c) active role play is the Mg-d orbital at 0.03 eV, secondly, Mg-p orbital at 0.02 eV near the Fermi level in the VBM region, and CBM shows a huge bandgap difference. Lastly, Fig. 4(d), active role play is the O-p orbital at 2.07 eV, secondly, the O-PX + PY orbital at 1.40 eV, thirdly, the O-PZ orbital at 0.59 eV in the VBM region and the CBM region, a huge bandgap exist there.

**Fig. 5:**
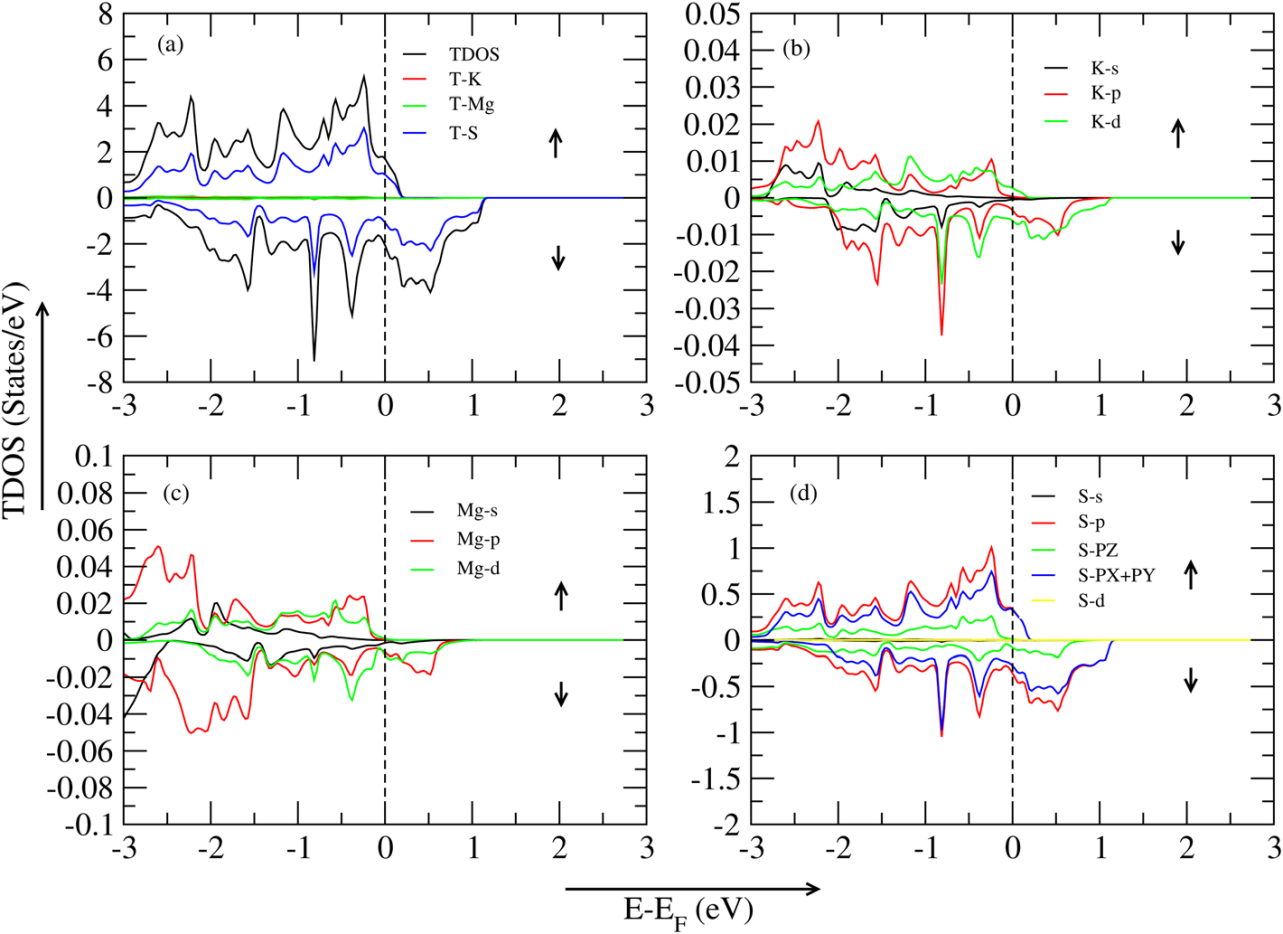
TDOS and PDOS of the KMgS_3_ compound with WC–GGA: (a) TDOS, (b) PDOS for K atoms, (c) PDOS for Mg atoms, and (d) PDOS for S atoms.

**Fig. 6:**
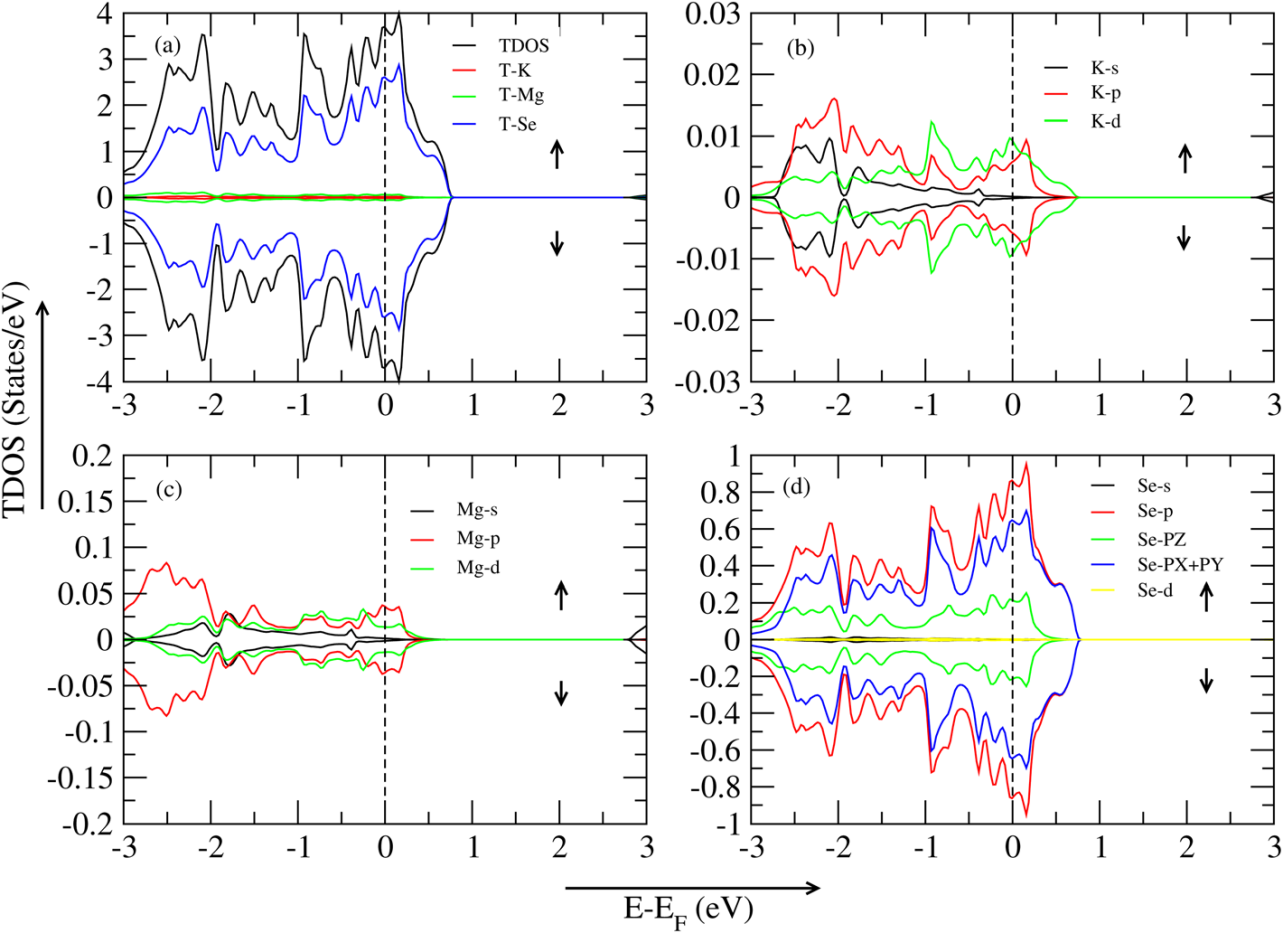
TDOS and PDOS of the KMgSe_3_ compound with WC–GGA: (a) TDOS, (b) PDOS for K atoms, (c) PDOS for Mg atoms, and (d) PDOS for Se atoms.

In this section, all graph is taken by the WC-GGA approximation method. Fig. 5(a) represents the TDOS (total density of states) of the KMgS_3_ compound. The material represents the metallic nature in both spin-up/spin-down states. The graph illustrates that the spin-up region makes a significant contribution to TDOS (KMgS_3_) at 4.94 eV, and secondly, the sulfur atom shows an active role in this region at around 2.85 eV of VBM and CBM region, no peak is shown in this graph region. In the spin-down region, there is no bandgap difference between them, so the material shows a metallic nature. In Fig. 4(b), spin-up active role play is the K-p hybrid orbital near to the Fermi level of 0.009 eV in VBM, and in the other part, CBM, a huge bandgap difference shows them, and spin-down is the metallic state. In Fig. 4(c), active role play is the Mg-p orbital at 0.0214 eV, secondly, Mg-d orbital at 0.009 eV near to Fermi level in the VBM region and CBM shows a metallic nature. Lastly, in Fig. 4(d), active role play is the S-p orbital at 0.05 eV, secondly, the S-PX + PY orbital at 0.69 eV, thirdly, the S-PZ orbital at 0.21 eV in the VBM region and CBM region, a huge bandgap exists there. In this section, all graphs are taken by the WC-GGA approximation method. Fig. 6(a) represents the TDOS (total density of states) of the KMgSe_3_ compound, and the material is metallic in both spin-up/spin-down states. The significant contribution on both sides of VBM/CBM is the T-Se atom. In Fig. 6(b), in both spin-up/spin-down states, the K-d orbital significant contribution is shown. In Fig. 6(c), Mg-p shows an active role in both spin-up/spin-down states. Lastly, the Fig. 6(d), Se-p (firstly), Se-PX + PY (secondly), and Se-PZ (thirdly) hybrid orbitals show a significant contribution in both states of the spin-up/spin-down region.

The magnetism of the KMgX_3_(X = O, S, Se) system presents an unconventional and compelling case, as it arises without the presence of transition metals, which are typically essential for magnetic behaviour due to their partially filled d or f orbitals. In KMgX_3_ compounds, magnetism is primarily attributed to the anions (O, S, Se) through their partially filled p-orbitals, leading to what is known as p-orbital magnetism or d^°^ magnetism. First-principles calculations reveal a spin-polarized electronic structure, indicating that these materials may exhibit a ferromagnetic ground state. The unpaired p-electrons, particularly those on the oxygen or chalcogen atoms, are responsible for the local magnetic moments. The strength and nature of the magnetic ordering vary across the series, influenced by the size and electronegativity of the anion. Oxygen, being the most electronegative and smallest, tends to localize the p-electrons more strongly, potentially enhancing magnetic interactions, while sulphur and selenium, being larger and less electronegative, may lead to broader p-bands and weaker localization of magnetic moments. Additionally, the indirect exchange interactions (possibly super exchange or p–p hybridization mechanisms) between neighbouring anions mediated through nonmagnetic Mg atoms play a critical role in stabilizing the ferromagnetic ordering. This unique magnetic behaviour makes the KMgX_3_ compounds promising candidates for spintronic applications, especially considering their transition-metal-free and potentially environmentally benign nature.

### 3.4 Elastic properties

When describing the materials in practical applications, efficient elastic constants are a helpful tool. The material's reactivity to external forces and structural stability are characterized by its elastic properties. Three distinct components make up the elastic stiffness tensor for KMgX_3_(X = O, S, and Se) compounds with cubic symmetry: *C*_11_, *C*_12_, and *C*_44_.^29,30^ The calculated values of elastic constants for perovskite chalcogenides are given in Table 3. The Born stability criteria, sometimes referred to as the mechanical stability conditions for these materials, must be met by the elastic stable compound KMgX_3_(X = O, S, and Se).^31–33^ It's clear from Table 3 that, the computationally calculated values of elastic constants for KMgO_3_, KMgS_3_ and KMgSe_3_ materials fulfilled the Born stability criteria, and these values are also in good agreement with other available results.^34,35^ Other critical parameters of mechanical properties can be determined by using the elastic constants. Mechanical parameters, such as shear modulus (*G*_H_), bulk modulus (*B*_0_), Young's modulus (*Y*), Poisson's ratio (*ν*), Pugh's ratio (*B*/*G*), Cauchy's pressure (*C*), and anisotropic ratio (*A*), are mentioned in Table 4, respectively. Moreover, in the theory of elasticity, several parameters are used to describe how materials deform under applied forces. Among the most fundamental are the Lame parameters, denoted by *λ* (lambda) and *μ* (mu). These parameters are central to the generalized Hooke's law for isotropic materials. The parameter *μ*, also known as the shear modulus or the second Lame parameter, characterizes the material's response to shear stress, that is, how it deforms when forces are applied parallel to its surface. The other Lame parameter, *λ*, works in tandem with the other to describe volumetric and shear behaviour in materials under stress. Along with these, functional parameters, *C* generally refers to the elastic stiffness tensor, often written as *C*_ijkl_ in tensor notation. This tensor governs the relationship between stress and strain in the most general form of Hooke's law, especially for anisotropic materials, where different directions in the material respond differently to stress. All these parameters are represented in Table 3.

**Table 3:** The computational elastic stiffness constants (*C*_ij_) and Cauchy's pressure (*C*_p_) of KMgX_3_(X = O, S and Se) materials

Compound	*C*_11_	*C*_12_	*C*_44_	*C*_p_
KMgO_3_	183.74	38.84	21.765	17.074
Other^28^	121.54	32.57	9.0363	23.535
KMgS_3_	50.01	8.99	1.54	7.448
KMgSe_3_	73.70	16.64	36.42	−19.782

**Table 4:** The calculated mechanical parameters for KMgX_3_(X = O, S and Se) compounds

Compound	KMgO_3_	KMgS_3_	KMgSe_3_
*G*_V_ (GPA)	42.04	9.12	33.26
*G*_R_ (GPA)	30.22	2.44	32.79
*G*_H_ (GPA)	36.13	5.79	33.03
*B*_o_	87.14	22.66	35.66
*Y*	108.6	24.14	76.12
*B*/*G*	2.41	3.92	1.08
*C*′′ Cauchy's pressure	17.07	7.45	−19.78
*λ* Lame's first parameter	59.11	16.58	13.48
*μ* shear modulus	42.04	9.13	33.26
*ν* Poisson's ratio	0.29	0.32	0.14
*ζ* kleinman parameter	0.41	0.37	0.43
*A* anisotropic ratio	0.30	0.07	1.28
*C*′ shear constant	72.45	20.51	28.53

Another important parameter of a mechanical properties can be determined by using the elastic constants. The mechanical parameters such as shear modulus (*G*_H_), bulk modulus (*B*_o_), Young's modulus (*Y*), Poisson's ratio (*ν*), Pugh's ratio (*B*/*G*), Cauchy's pressure (*C*′′) and anisotropic ratio (*A*) which is mentioned in Table 4, respectively. The calculated average Hill's shear modulus (*G*_H_), finding the plastic deformations brought by shear stress. So *G*_H_ tell us the material's hardness and equal to arithmetic mean of the Voigt (*G*_v_) and Reuss (*G*_R_) moduli. The mathematically expression for *G*_H_ is:^36^2
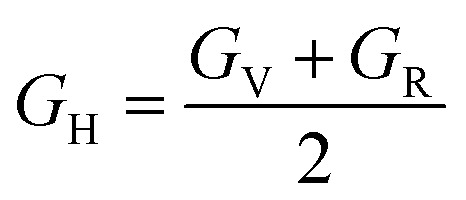
where3
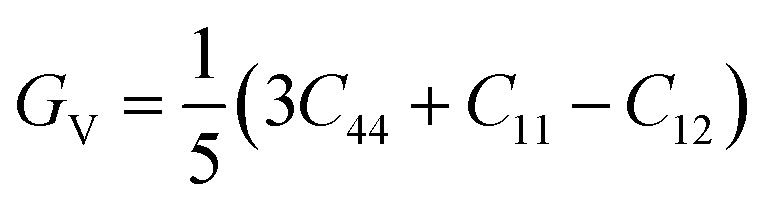
and4
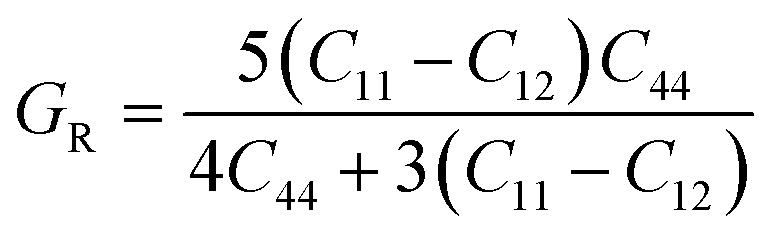


In Table 4, the value of *G*_H_ reveals that, KMgO_3_ exist the highest value (great hardness) of *G*_H_ (36.13 GPa) and the lowest value (less hardness) of *G*_H_ (5.79 GPa). Young's modulus tell us the stiffness of a compound.^37^ Using the following formula, Young's modulus may be calculated from the *G*_V_ and *B*_o_.5
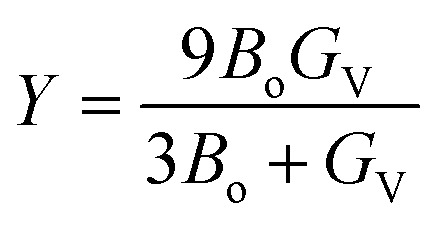


KMgO_3_ has largest value of Young modulus which is more stiffer than the other materials in Table 4 under study. These materials are harder to break because their Young's moduli are higher than their bulk and shear moduli. Understanding Pugh's ratio (*B*/*G*) is crucial to comprehending the ductile/brittle character of materials. If the ratio of *B*/*G* is more than 1.75, the material is deemed ductile; otherwise, it is deemed brittle.^38^ For the KMgX_3_ pervoskites under study, the *B*/*G* ratio is shown in Table 4, and the values are one is below and other are above than 1.75. So KMgO_3_ and KMgO_S_ are ductile and KMgSe_3_ is brittle due to less than value of 1.75. An further indicator of the materials' brittle and ductile nature is the Cauchy pressure (*C*′′ = *C*_12_ − *C*_44_).^39^ A positive or negative *C*′′ value denotes brittle or ductile behavior. The positive value of KMgO_3_ and KMgS_3_ show ductile nature, and the negative value of KMgSe_3_ brittle nature. Metallic compounds typically exhibit delocalized electrons arising from partially filled energy bands, which leads to non-directional metallic bonding. This delocalization not only results in high electrical conductivity but also allows atomic planes to slide over one another more easily, contributing to a generally ductile mechanical response. In contrast, covalent or ionic compounds are characterized by localized electrons in fully occupied bands and strong directional bonding, which restricts atomic movement and typically results in lower electrical conductivity and brittle mechanical behaviour. Thus, the absence of a band gap or the presence of partially filled bands is often correlated with ductility. However, this correlation is not absolute, particularly in complex oxides and chalcogenides like KMgSe_3_, where factors such as bonding anisotropy, low density of states at the Fermi level, or unfavourable elastic properties can lead to brittle behavior despite metallic conductivity. In metallic materials, strong bonding anisotropies such as those found in layered structures or materials with directional Mg–Se bonds can significantly restrict dislocation motion, which is crucial for plastic deformation. When the bonding character is partially ionic or covalent rather than fully metallic, the material may still exhibit brittle behaviour under mechanical stress, despite having a metallic electronic structure. Additionally, the density of states (DOS) at the Fermi level plays a vital role; a low DOS implies fewer free carriers available to screen lattice distortions, which can further reduce ductility. In this case of KMgSe_3_, it may appear metallic in terms of its band structure. Still, if the carrier density and bonding softness are insufficient, it may not be “metallic enough” to enable ductile behaviour.

Furthermore, mechanical stability and ductility are often evaluated using DFT-derived mechanical parameters such as Pugh's ratio (*B*/*G*) and Cauchy pressure (*C*_12_ − *C*_44_). According to Pugh's criterion, a material is considered ductile if *B*/*G* < 0.57 and brittle if *B*/*G* > 0.57. Similarly, a positive Cauchy pressure suggests a ductile nature, whereas a negative value indicates brittleness. Therefore, if KMgSe_3_ exhibits a high *B*/*G* ratio or negative Cauchy pressure, this would explain its brittle mechanical behavior, even in the presence of metallic conductivity. Despite KMgSe_3_ being metallic, it is mechanically less ductile than KMgS_3_, as shown by its higher *G*/*B* ratio and lower bulk modulus. However, both have positive Cauchy pressures, indicating some ductility. KMgSe_3_'s ductility might be reduced further under strain or defects, aligning with the observed tendency toward brittleness in practical conditions, particularly due to bonding anisotropy or low density of states at the Fermi level.

Anisotropic ratio (*A*) is the way to quantify the intensity of various characteristics in different directions and given as:6
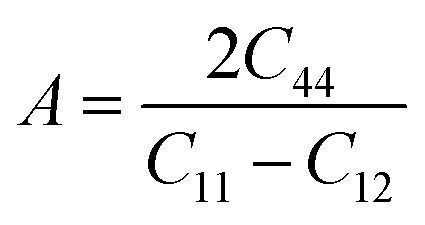
where *A* become unity that mean a material is isotropic; otherwise it's anisotropic nature. Elastic anisotropy is important in the utilization of engineering materials and is closely linked to the likelihood of induced microcracks in a material. Table 4 shows that the anisotropic ratio value is not equal to unity. The departure of *A* from unity indicates that all of these compounds are elastically anisotropic and that their characteristics vary in various directions. A parameterization of the elastic moduli for homogeneous isotropic material is established by the two parameters *λ* and *μ*, which are referred to as Lame's first and second constants, respectively. Poisson's ratio and young modulus may be used to compute *λ* and *μ*:7
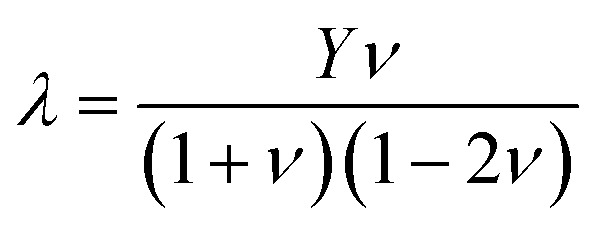
and8
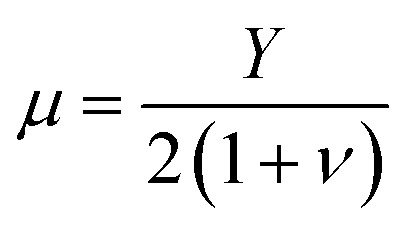


Table 4 lists their values for KMgX_3_ compounds (where X = O, S, and Se). The values of *λ* = *C*_12_ and *μ* = *C*′ are for isotropic molecules. Considering that the compounds under investigation are highly anisotropic and do not satisfy the criteria for isotropic compounds. Poisson's ratio (*ν*), which is balanced by resistance to shape change, indicates the material's resistance to volume change.^40^ The following relation is used to compute Poisson's ratio, or *ν*:9
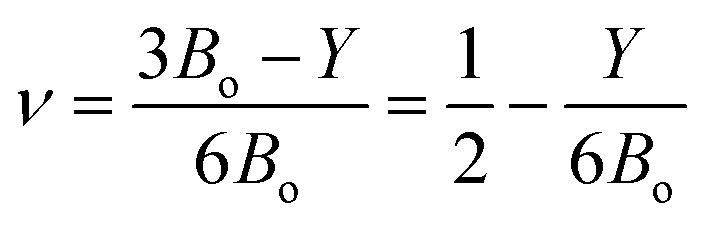


The value of *ν* for ductile (brittle) character is 
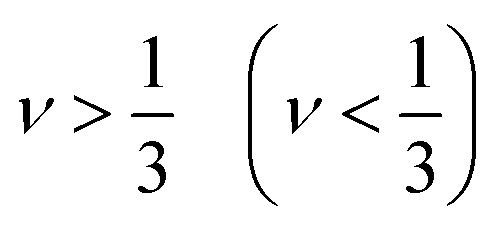
. It's clear from the Table 4 that, KMgSe_3_ exist brittle and other two are ductile but its value are falls below than 1/3. Kleinman parameter (*ζ*) quantify the internal strain of a material and describe the proportion of bond bending to bond stretching.^41^ To obtained the value of *ζ* we used the following relation:^42^10
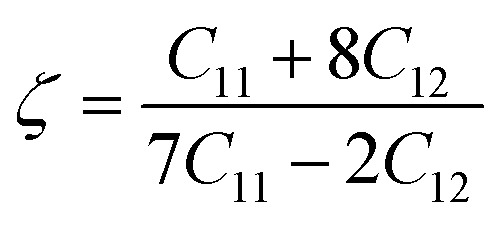


For *ζ* = 1 enables the bond bending constraints, while *ζ* = 0 enables the bond stretching constraints, respectively. The calculated for KMgSe_3_ (*ζ* = 0.43) predict that bond stretching is dominant in this compound. For other compounds KMgX_3_ (X = O and S), bond bending is dominant. An important parameter shear constant *C*′ define the dynamical stability against tetragonal distortion of a material and calculated by:11
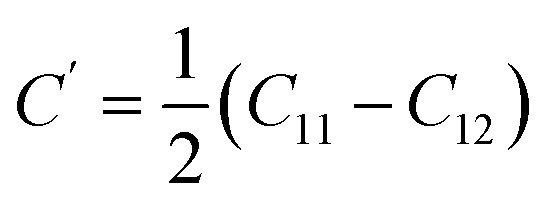


**Fig. 7:**
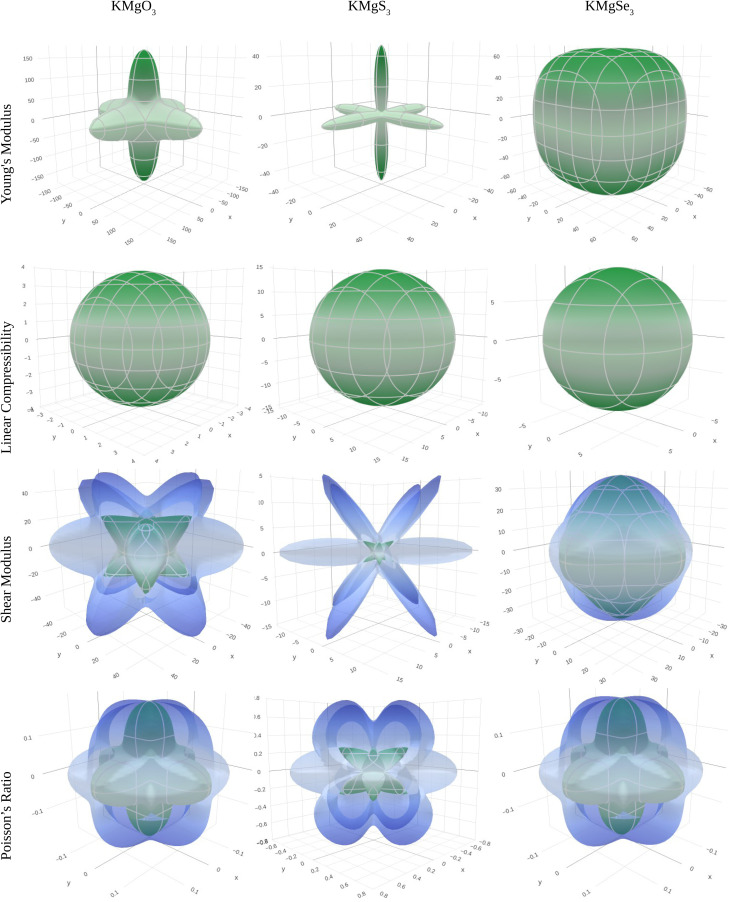
Three-dimensional (3D) spatial dependence of Young's modulus (*Y*), linear compressibility, shear modulus (*G*), and Poisson's ratio for KMgX_3_ (X = O, S, and Se) compounds, obtained using the Elate program.

The positive value of shear constant *C*′ (*C*′ > 0) fulfill the required criteria (Table 4). Hence, all the compounds under study are dynamically stable materials. In order to graphically represent the anisotropic and elastic characteristics of the materials under study, three-dimensional (3D) contour plots were created using the Elate program^43^ for (a) Young's modulus (*Y*), (b) linear compressibility, (c) shear modulus (*G*), and (d) Poisson's ratio. For KMgO_3_, KMgS_3_, and KMgSe_3_, respectively, these 3D representations are shown in Fig. 4. While linear compressibility is largely spherical among the characteristics shown, the other elastic constants show notable departures from spherical symmetry, highlighting the compounds' strong anisotropic nature (Fig. 7).

### 3.5 Phonon dispersion curves and thermal stability

**Fig. 8:**
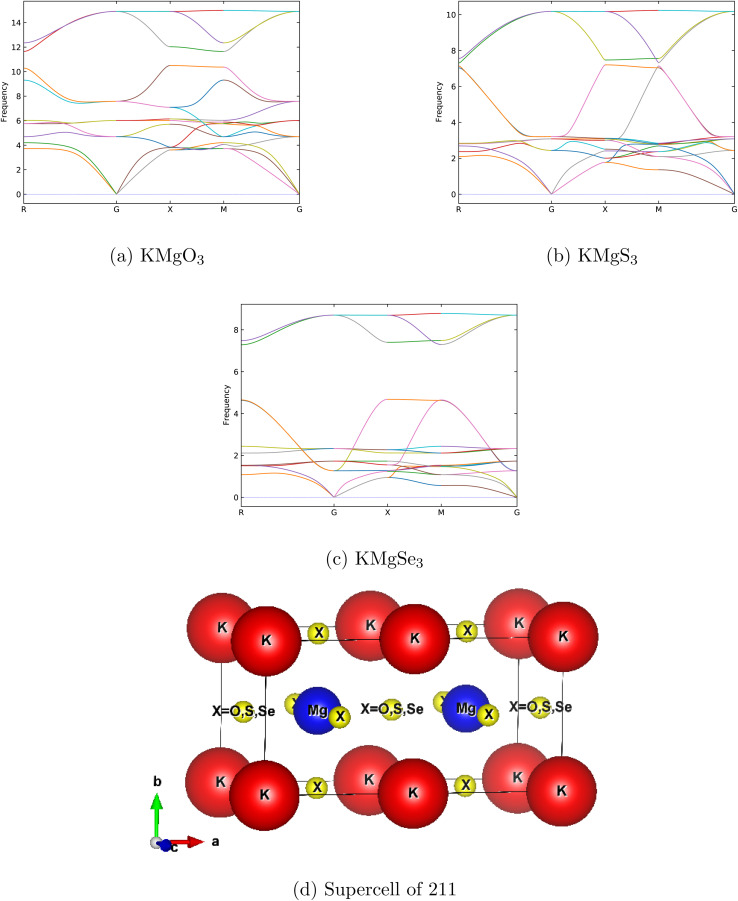
Phonon dispersion curves for KMgX_3_(X = O, S and Se) materials: (a) KMgO_3_, (b) KMgS_3_, (c) KMgSe_3_, and (d) 2 × 1 × 1 supercell diagram.

**Fig. 9:**
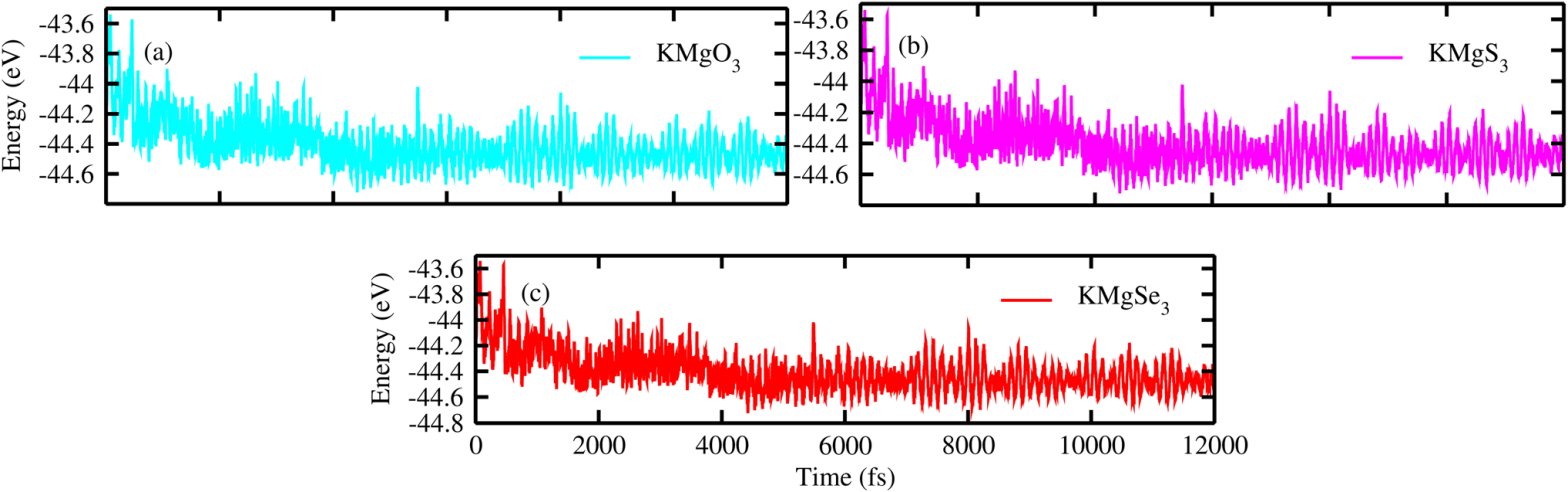
Thermal stability of KMgX_3_ (X = O, S, and Se) compounds *via* AIMD simulations: (a) KMgO_3_, (b) KMgS_3_, and (c) KMgSe_3_.

To verify the dynamical stability of KMgX_3_ (X = O, S and Se) ternary alkali-earth metal chalcogenides perovskite in *Pm*3̄*m* symmetry, the phonon dispersion curve of KMgO_3_, KMgS_3_ and KMgSe_3_ are shown in Fig. 8(a, b and c), respectively. It's reveals from the figures that, no negative frequencies are shown and the materials are dynamically stable in their cubic phase. KMgX_3_ compounds consists of five atoms in their unit cell so their results includes 15 numbers of phonon branches in KMgO_3_, KMgS_3_ and KMgSe_3_ thats includes 3 acoustic and 12 optical branches. Further investigation reveals that, *ab initio* molecular dynamic simulations (AIMD)^44^ are performed to check the thermal stability of KMgX_3_ chalcogenides perovskite, at 300 K the simulation was run for 12 ps (12 000 fs) with a time step of 1 fs, as depicted in Fig. 9(a, b and c). It's evident from the figures that no broken bonds are observed and all of these compounds remains thermally stable without the structural distortion in their respective positions (Fig. 8d).

## 4 Conclusion

KMgX_3_ (X = O, S, Se) perovskites were analyzed using DFT, revealing their stable cubic phase with *Pm*3̄*m* symmetry and distinct electronic properties. KMgO_3_ was found to be a semiconductor with a bandgap of 7.323 eV, while KMgS_3_ and KMgSe_3_ exhibited metallic behavior within the WC-GGA functional. However, using the TB-mBJ potential, the bandgaps of KMgO_3_ and KMgS_3_ increased to 11.102 eV and 5.391 eV, respectively. Using Born criteria, mechanical stability was verified, exhibiting brittleness in KMgSe_3_ and ductility in KMgO_3_/S_3_. The resilience of their dynamics was confirmed by phonon and thermal simulations. The results show that KMgX_3_, especially KMgO_3_, has potential in spintronics and optoelectronics. For their useful applications in advanced materials to be realized, more experimental confirmation is necessary.

## Conflicts of interest

There are no conflicts of interest to declare.

## Data Availability

The data supporting the findings of this study are available within the article and its SI materials. Additional datasets generated during this computational investigation, including input files for WIEN2k simulations, optimized crystal structures, and raw output files for electronic/elastic property calculations, are available from the corresponding author (Mazhar Haleem Awan, mazharhaleem7@gmail.com) upon reasonable request. All DFT calculations were performed using open-source computational methods implemented in the WIEN2k package (version 23.1). Phonon dispersion data were obtained using the PHONOPY code, and visualization was done with VESTA software. Relevant computational parameters and methodology are fully described in the Methods section of this manuscript.
